# Transvaginal ultrasound-guided aspiration of an anterior sacral meningocele masquerading as a hydrosalpinx, resulting in abscess formation

**DOI:** 10.1259/bjrcr.20160037

**Published:** 2016-09-03

**Authors:** Susan Jawad, Vivian Ejindu, Declan Johnson, Mohammad Ali Shah, Maaz Ali Abbasi, Kamal Ojha, Nikolaos Papadakos

**Affiliations:** ^1^Department of Clinical Radiology, St. George's Hospital NHS Foundation Trust, London, UK; ^2^Department of Neuroradiology, St. George's Hospital NHS Foundation Trust, London, UK; ^3^Department of Obstetrics and Gynaecology, St. George's Hospital NHS Foundation Trust, London, UK

## Abstract

Anterior sacral meningoceles (ASMs) have a recognized association with a number of connective tissue disorders, including Marfan’s syndrome, neurofibromatosis Type 1 and Ehlers–Danlos syndrome. We present the case of a patient with Marfan’s syndrome and ASMs who was referred to gynaecology owing to dysmenorrhoea and left-sided pelvic pain radiating to the left leg. A transvaginal ultrasound scan (TVUS) detected a left pelvic cystic tubular structure, attributed to a hydrosalpinx, which, in retrospect, likely corresponded to the ASM. The patient went on to have TVUS-guided drainage of this cystic structure, resulting in an ASM abscess. It is difficult to distinguish ASM from the vastly more common hydrosalpinx using TVUS alone, and in patients with an atypical appearing posteriorly positioned cystic pelvic lesion or in the presence of underlying conditions known to be associated with ASMs, MRI should be considered before any interventional procedure to drain the suspected hydrosalpinx transvaginally. The patient was successfully treated using a minimally invasive CT-guided posterior trans-sacral drainage technique.

## Clinical presentation

**Figure 1.**
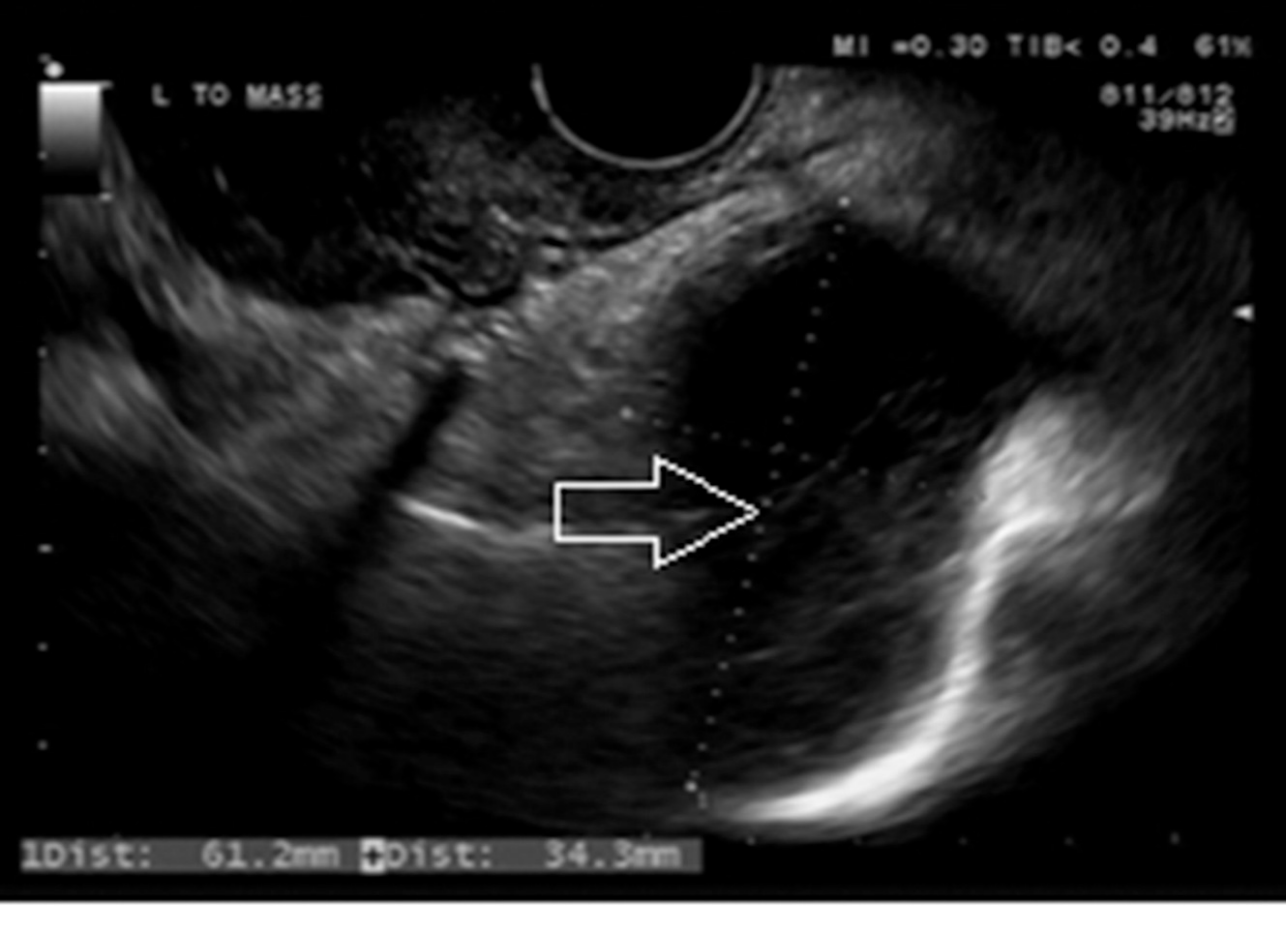
Transvaginal ultrasound scan showing the predominantly cystic left pelvic lesion (arrow) interpreted as a left-sided hydrosalpinx.

A 42-year-old patient with Marfan’s syndrome and a metallic aortic valve replacement presented to the gynaecologists with left-sided pelvic and back pain, and dysmenorrhoea. Because of the patient's age, a hysteroscopy and transvaginal ultrasound scan (TVUS) were arranged. Pipelle biopsy from the hysteroscopy was negative; however, the TVUS detected a cystic tubular lesion in the left pelvis, which was attributed to a hydrosalpinx (Figure 1). The left pelvic pain continued over the next 4 years and was later associated with radiation down the left leg.

Owing to the presence of a metallic heart valve necessitating anticoagulation and other significant comorbidities, the patient was deemed to have a high anaesthetic and surgical risk, and therefore, the usually preferred treatment for suspected hydrosalpinx of laparoscopy and salpingectomy was not pursued. Instead, the clinical team opted for the less invasive technique of TVUS-guided hydrosalpinx aspiration. This was performed on two separate occasions as a day care procedure. After both aspirations, the patient started suffering from a severe frontal headache that led to emergency admission and a CT scan of the head. The investigations were all unremarkable and the headache was attributed to migraine. On both occasions, the patient was discharged with conservative management only.

Owing to the ongoing symptoms, a third aspiration was performed and the patient remained in the hospital to restart warfarin and establish a therapeutic international normalized ratio post aspiration. During this interval, the patient became septic and complained of increasing pelvic and back pain. She was rescanned transvaginally, revealing reaccumulation of the suspected left-sided hydrosalpinx. As the inflammatory markers continued to increase, the working clinical diagnosis was that of a developing tubo-ovarian abscess.

## Investigations/imaging findings

**Figure 2.**
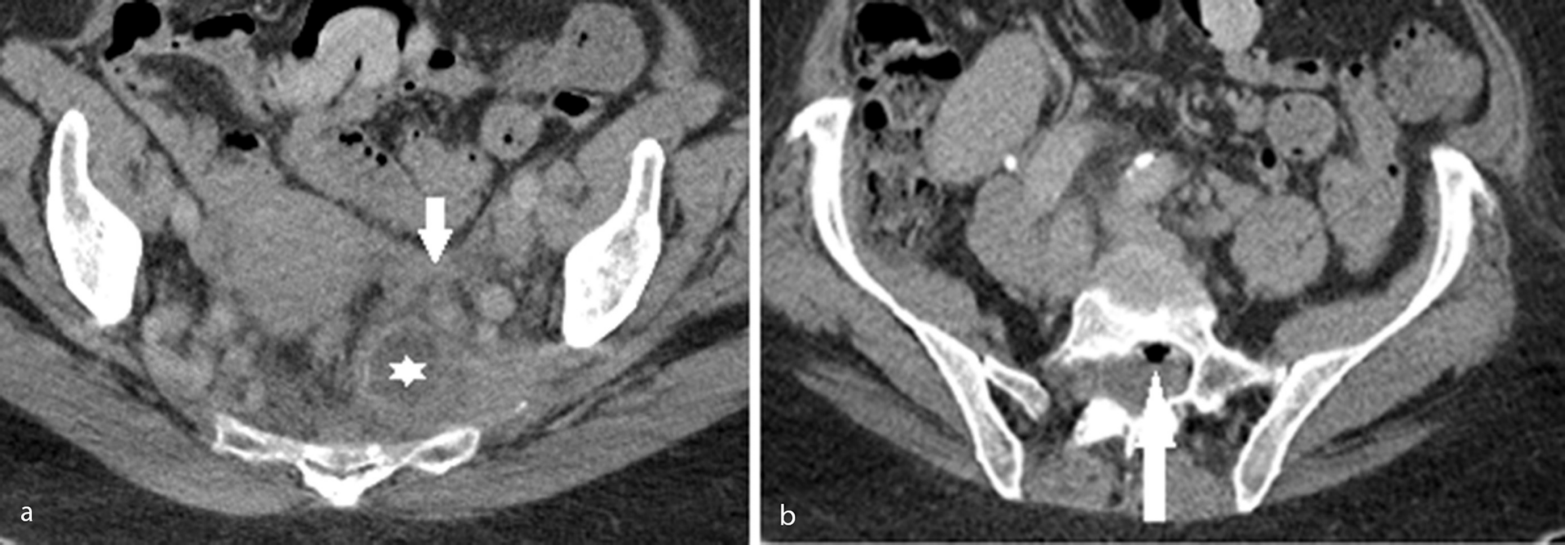
(a) Infected left-sided anterior sacral meningocele (star), which is discrete from the normal calibre left adnexa (arrow). (b) Gas-containing (arrow), enhancing sacral cyst, which is consistent with an abscess within an infected sacral cyst.

Owing to the worsening clinical state, a CT scan of the abdomen and pelvis was performed, revealing a thickened, enhancing left-sided anterior sacral meningocele (ASM) (Figure 2a), which was in direct communication with a thickened, enhancing and gas-containing sacral cyst (Figure 2b); appearances were in keeping with an infected ASM and sacral cyst containing an abscess. No hydrosalpinges or abnormal adnexal lesions were present on the CT scan.

**Figure 3.**
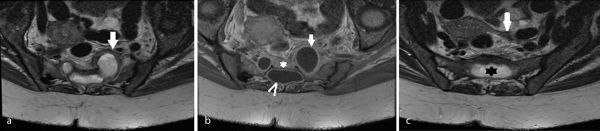
(a) Axial *T*_2_ weighted sequence through the pelvis at the level of bilateral ASMs showing thickening of the ASMs and sacral cyst (arrow). (b) Axial contrast-enhanced *T*_1_ weighted sequence through the pelvis at the level of bilateral ASMs showing enhancement of the ASM (arrow) and sacral cyst (arrowhead), and inflammatory stranding in the presacral soft tissues (star). (c) Axial *T*_2_ weighted sequence through the pelvis at the level of the sacral cyst (star). The left adnexa is of normal calibre and does not show any increased *T*_2_ signal to suggest tubal fluid or hydrosalpinx (arrow). ASM, anterior sacral meningocele.

Subsequent MRI of the whole spine and pelvis showed similar findings, with inflammatory changes in the presacral soft tissues and a thickened, enhancing sacral cyst and an ASM protruding through the sacral foramina. This contained high and heterogeneous *T*_2_ signal material, later confirmed to be pus upon drainage (Figures 3a–c). The remaining spinal imaging was unremarkable with no evidence of any inflammatory change affecting the thecal sac or spinal canal contents.

## Treatment

The patient was initially treated with empirical intravenous antibiotics. Despite this, her inflammatory markers deteriorated. After discussion at the spinal multidisciplinary meeting and in light of the anaesthetic risk, it was decided that a CT-guided drainage should be performed using a posterior sacral transosseous approach, rather than surgical drainage.

**Figure 4.**
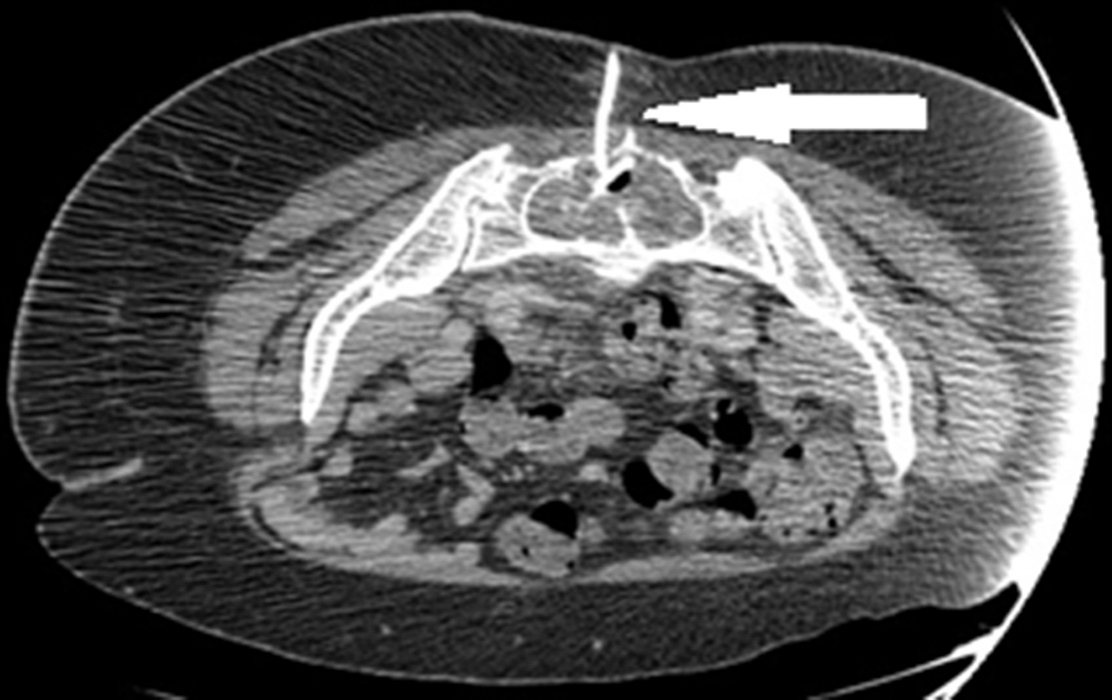
CT-guided drain insertion. The pigtail drain (arrow) has been successfully sited in the abscess within the sacral cyst, facilitated by the markedly thinned sacral lamina, permitting passage of a standard drainage access needle.

The longstanding sacral cyst had resulted in marked thinning of the sacral cortex. As such, a 5.0 French Kellet drainage access needle (Cook® Medical, Bloomington, IN, USA) was sufficient to be passed transosseously through the thinned posterior mid-sacrum into the sacral cyst. After accessing the sacral cyst, a guide wire was advanced through the sheath of the drainage needle and an 8-French locking pigtail drain was introduced over the guide wire using a Seldinger technique (Figure 4). Approximately 80 ml of frank pus was aspirated and the final drain position was confirmed using repeat intraprocedural imaging. Following this, the patient made a rapid recovery, with resolution of the sepsis and inflammatory markers. The drain was removed at day 2 post procedure, having drained no further significant pus or fluid. The patient was discharged on day 12 post procedure and went on to make a good clinical recovery with no recurrent documented symptoms at her 6-month outpatient follow-up.

## Discussion

Extradural sacral cysts arise from the caudal tip of the dural sac. As a result of mass effect and cerebrospinal fluid (CSF) pulsations, likely owing to the presence of a valve mechanism causing intermittent surges of CSF pressure, erosion of both the anterior and posterior bony sacrum can occur.^1^ In some cases, the enlarged sacral cyst can bulge through an anterior bony defect or neural foramina as an ASM.^2^

Marfan’s syndrome is a hereditary multisystem connective tissue disorder, which is inherited in an autosomal dominant manner. Musculoskeletal manifestations are common and diverse, and include ASM and dural ectasia of the spine.^3,4^ Other conditions also associated with ASM include neurofibromatosis Type 1 and Ehlers–Danlos syndrome.^5,6^

The original presenting symptoms in this patient with dysmenorrhoea, and left-sided pelvic and back pain radiating down the leg are recognized symptoms of ASM.^7–9^ In light of the frontal headache after each of the two previous TVUS-guided aspirations of the suspected hydrosalpinx, it is likely the ASM was mistaken for a hydrosalpinx ultrasonographically and CSF was aspirated.

It would be very difficult to distinguish an ASM from a hydrosalpinx ultrasonographically, as both would appear as blind-ending cystic structures within the pelvis.

Transvaginal interventional procedures have the disadvantage of only being semisterile and although generally reported as being a low-risk procedure for adnexal lesions,^10^ in this case, TVUS-guided aspiration of the ASM, masquerading as a hydrosalpinx, resulted in sepsis.

In patients at risk of having ASM owing to connective tissue disorders, such as Marfan’s syndrome, if hydrosalpinx is suspected ultrasonographically, MRI should be considered to confirm the spinal and sacral anatomy and elucidate the nature of the cystic lesion prior to proceeding to any interventional procedure.

ASM infection and abscess formation with a range of aetiologies is well recognized. In all reported cases, open surgical drainage was undertaken as definitive treatment. To our knowledge, this is the first reported case in the literature whereby the technique of minimally invasive CT-guided trans-sacral drainage was used to successfully treat an infected ASM and sacral cyst abscess.

## Learning points

1. Marfan’s syndrome and other connective tissue diseases can be associated with ASM.
2. Distinguishing an ASM from a hydrosalpinx in a female patient by TVUS is challenging, as both appear as blind-ending pelvic cystic structures.
3. If intervention for suspected hydrosalpinx is being considered in a patient with a condition predisposing them to ASMs, such as Marfan's syndrome, a pelvic MRI should be performed to assess the gynaecological anatomy and confirm the presence of hydrosalpinx before proceeding with a transvaginal approach to aspiration.
4. Patients with Marfan's syndrome often have cardiovascular complications and therefore present a significant anaesthetic risk for surgical procedures. CT-guided trans-sacral drainage of ASM abscesses in the presence of a sacral cyst is a potential treatment as an alternative to surgical drainage for potentially high-risk anaesthetic cases.

## Consent

Informed consent has been obtained for the images and data in publishing this case and is held on record.

## References

[1] Nabors MW, Pait TG, Byrd EB, Karim NO, Davis DO, Kobrine AI, et al Updated assessment and current classification of spinal meningeal cysts. J Neurosurg 1988; 68: 366–77. 3343608 10.3171/jns.1988.68.3.0366

[2] Villarejo F, Scavone C, Blazquez MG, Pascual-Castroviejo I, Perez-Higueras A, Fernandez-Sanchez A, et al Anterior sacral meningocele: review of the literature. Surg Neurol 1983; 19: 57–71. 6828997 10.1016/0090-3019(83)90212-4

[3] Nallamshetty L, Ahn NU, Ahn UM, Nallamshetty HS, Rose PS, Buchowski JM, et al Dural ectasia and back pain: review of the literature and case report. J Spinal Disord Tech 2002; 15: 326–9. 12177551 10.1097/00024720-200208000-00012

[4] De Paepe A, Devereux RB, Dietz HC, Hennekam RC, Pyeritz RE. Revised diagnostic criteria for the Marfan syndrome. Am J Med Genet 1996; 62: 417–26. 8723076 10.1002/(SICI)1096-8628(19960424)62:4<417::AID-AJMG15>3.0.CO;2-R

[5] Sahin N, Genc M, Kasap E, Solak A, Korkut B, Yilmaz E, et al Anterior sacral meningocele masquerading as an ovarian cyst: a rare clinical presentation associated with Marfan syndrome. Clin Pract 2015; 5: 752. 26236457 10.4081/cp.2015.752 PMC4500879

[6] Strand RD, Eisenberg HM. Anterior sacral meningocele in association with Marfan’s syndrome. Radiology 1971; 99: 653–4. 5578713 10.1148/99.3.653

[7] Polat AV, Belet U, Aydin R, Katranci S Anterior sacral meningocele mimicking ovarian cyst: a case report. Med Ultrason 2013; 15: 67–70. 23486628 10.11152/mu.2013.2066.151.avp1asm2

[8] Erdogmus B, Yazici B, Ozdere BA, Safak AA. Anterior sacral meningocele simulating ovarian cyst. J Clin Ultrasound 2006; 34: 244–6. 16673368 10.1002/jcu.20198

[9] Voyvodic F, Scroop R, Sanders RR. Anterior sacral meningocele as a pelvic complication of Marfan syndrome. Aust N Z J Obstet Gynaecol 1999; 39: 262–5. 10755797 10.1111/j.1479-828x.1999.tb03390.x

[10] Scanlan KA, Propeck PA, Lee FT. Invasive procedures in the female pelvis: value of transabdominal, endovaginal, and endorectal US guidance. Radiographics 2001; 21: 491–506. 11259711 10.1148/radiographics.21.2.g01mr21491

